# Infectious consequences of antimicrobial change from vancomycin-piperacillin/ tazobactam to vancomycin-cefepime in the prevention of AKI during orthotopic heart transplantation

**DOI:** 10.1186/s12879-026-12788-8

**Published:** 2026-03-21

**Authors:** Ashley L. Golbus, Elaine Park, Courtney E. Harris, Syed Quadri, Rupak Mukherjee, Arman Killic, Ryan J. Tedford, Blaithin A. McMahon

**Affiliations:** 1 Department of Medicine, Medical University of South Carolina, Charleston, SC USA; 2 College of Medicine, Medical University of South Carolina College of Medicine, Charleston, SC USA; 3 Division of Infectious Diseases, Department of Medicine, Medical University of South Carolina, Charleston, SC USA; 4 Division of Cardiothoracic Surgery, Department of Surgery, Medical University of South Carolina, Charleston, SC USA; 5 Division of Cardiology, Department of Medicine, Medical University of South Carolina, Charleston, SC USA; 6 Department of Medicine, Division of Nephrology, Medical University of South Carolina, 96 Jonathan Lucas Street, Suite 822 MSC 629, Charleston, South Carolina 29425 USA

**Keywords:** Orthotopic heart transplantation, Empiric antimicrobial, Post-operative infection, Acute kidney injury

## Abstract

**Background:**

Empiric antimicrobial selection in orthotopic heart transplantation (OHT) is critically important as post-operative infections are a significant cause of morbidity and mortality. While variability of antimicrobial selection exists between centers, both combinations of vancomycin and piperacillin-tazobactam (VPT) and vancomycin and cefepime (VC) are frequently used. VPT drug combination has been associated with increased risk of acute kidney injury (AKI) in multiple reports. Our study aimed to evaluate the infectious impact of changing the empiric antimicrobial selection from VPT to VC.

**Methods:**

This was a single-center study in patients who underwent OHT at the Medical University of South Carolina (MUSC) from 2015 through 2021 (n=120). As part of a quality improvement measure, the empiric intraoperative antimicrobial therapy was transitioned from VPT (n=48) to VC (n=72) on 6/20/2019, providing a prospective setting to investigate infectious outcomes.

**Results:**

A total of 10.4% of patients who received VPT had a positive culture within 30 days of OHT, compared to 9.7% of patients who received VC (p=0.781). All positive cultures were reviewed and deemed to be clinically significant infections. No significant difference in the site of culture positivity was identified between groups (p=0.487). Among patients who developed postoperative infection, there was a trend toward a longer time to infection in patients receiving VPT (median 28 days) than VC (median 14 days), p=0.08.

**Conclusions:**

Culture-positive infections within 30 days post-OHT were similar between patients receiving empiric intraoperative VPT and those receiving VC.

**Graphical Abstract:**

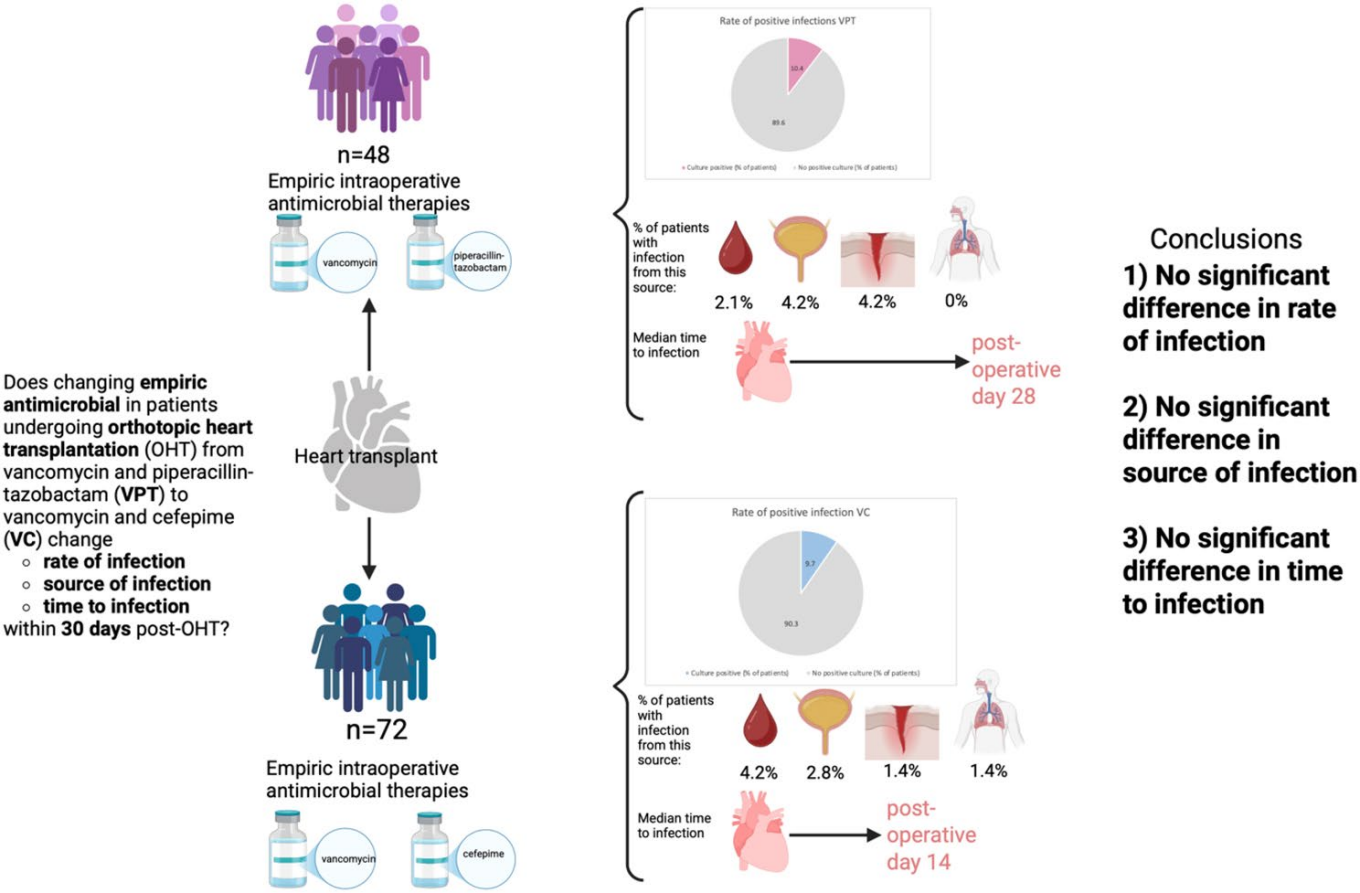

**Supplementary Information:**

The online version contains supplementary material available at 10.1186/s12879-026-12788-8.

## Background

Patients undergoing orthotopic heart transplantation (OHT) are at high risk for infection, requiring empiric broad-spectrum antibiotics peri-operatively. This population also has high rates of acute kidney injury (AKI) due to a combination of pre-, intra- and post-operative factors including comorbidities, hemodynamic instability, nephrotoxic medications, amongst others [1]. The KDIGO Clinical Practice guidelines for the management of AKI (2012) indicate the discontinuation of all nephrotoxic agents when possible [2]. In clinical practice, this has translated to stopping any nephrotoxic antibiotic and changing to less toxic antibiotics [2, 3]. The vancomycin and piperacillin-tazobactam (VPT) antimicrobial combination has been suggested to increase the risk of acute kidney injury [3]. Some cases of AKI associated with VPT use have generally been associated with mild stage 1 AKI, and less with more severe cases of stage 2/3 AKI or other patient-centered outcomes such as dialysis or mortality. In one large, recently published study using an intensive care unit (ICU) database of 335 hospitals involving over 35,000 patients, the authors reported an increased risk of VPT use limited to all stages of AKI [4]. In other studies, AKI associated with VPT may represent a pseudo-AKI; the observed increase in serum creatinine is suggested to occur as piperacillin-tazobactam binds and inhibits the tubular transporters OAT1 and OAT3, which secrete creatinine. This type of pseudo-AKI is not associated with any significant changes in alternative kidney function biomarkers, such as cystatin C or blood urea nitrogen, and is associated only with an isolated increase in serum creatinine [5]. We previously published a single-center prospective study involving adult patients who underwent OHT at the Medical University of South Carolina (*n* = 120), with the intervention of changing intraoperative microbial coverage from VPT to VC after the first 48 patients were transplanted. We concluded that transitioning empiric antimicrobial therapy from VPT to VC did not affect the incidence or severity of AKI or renal recovery rates [6].

Post-operative infection is a significant contributor to morbidity and mortality post-OHT, with infection occurring in up to 35% of these patients [7, 8]. While risk factors and characterization of these infections has been reported, data remains limited regarding the impact of empiric antimicrobial selection on infectious outcomes, particularly when comparing VPT to VC [7, 8]. Given concerns regarding VPT-associated nephrotoxicity and ongoing work in this area, it is important to determine whether changing empiric antimicrobial selection from VPT to VC alters infection risk. Therefore, our objective was to investigate whether a change in empiric intraoperative antimicrobial from VPT to VC changed the infectious outcomes post-OHT, including rates of infection, causative source, pathogens, or time to infection.

## Methods

### Study design

We performed a single-center study using data generated from a prior prospective analysis in adult patients undergoing OHT at the Medical University of South Carolina (MUSC), investigating the impact of changing empiric antimicrobial coverage from VPT to VC from 2015 to 2021 with the change from VPT to VC made in 2019. ^6^ All patients undergoing OHT at MUSC within this time frame were screened for eligibility. Exclusion criteria included peri-transplant extended spectrum beta-lactamase (ESBL) infection requiring meropenem. In the pre-intervention arm 48 patients were included and in the post-intervention arm 72 patients were included. Infections were classified as a positive bacterial culture (blood, urine, respiratory, or wound) for which the patient was treated with an antibiotic course. See our prior study utilizing this patient cohort for further inclusion criteria and demographic data [6].

Standard protocol included intraoperative dosing of vancomycin and piperacillin-tazobactam (pre-intervention) or vancomycin and cefepime (post-intervention). Dosing of piperacillin-tazobactam was standardized as 3.375 g IV then re-dosed every 2 h intraoperatively. Dosing of cefepime was standardized as 2 g IV, or renally dosed for creatinine clearance less than 60 mL/min, then re-dosed every 4 h for the duration the patient remained in the operating room; see Supplemental Fig. 1 for dosage details. While we did not have any patients with allergies precluding standard antimicrobial prophylaxis with VPT or VC, protocol directed consulting transplant infectious disease team and pharmD and dosing with vancomycin as standard in combination with Aztreonam 2 g IV. Antimicrobials were not continued beyond the intraoperative dosing unless the patient had a positive culture, positive donor culture or had a high degree of clinical suspicion for infection per transplant infectious disease consult team, at which time the antimicrobial choice was tailored accordingly.

The endpoints of the current study included the incidence of infection within 30 days of OHT or by time of discharge if this was within 30 days of OHT, source of infection, cultured organisms, and time to infection for patients with a positive culture. This timeframe was selected to focus specifically on early post-operative infections most likely to be influenced by peri-operative antimicrobial selection.

### Statistical analyses

Continuous variables are reported as medians and interquartile ranges and were analyzed via the Mann-Whitney test. Categorical variables were analyzed via Fisher’s exact tests. Statistical significance was determined by *p* < 0.05. Data was analyzed using the Stata software package (v17.0, College Station, TX).

## Results

### Baseline patient characteristics

Our previous study details baseline and intraoperative patient characteristics compared between VPT and VC groups [6]. To highlight specific variables most relevant to the present study, patients in the VPT group compared to the VC group were younger (median age of 50.0 years old [IQR 15.0 years] vs. 57.0 years [IQR 15.0 years]; *p* = 0.029) and had lower rates of induction immunosuppression (48% vs. 72%; *p* = 0.007). Other pre-operative and peri-operative characteristics were similar between groups. This included rates of medical therapy versus mechanical circulatory support (MCS) as bridge to transplant, with 27.1% of VPT patients and 43.1% of VC patients having MCS (*p* = 0.075). This also included sex distribution (29.0% vs. 37.5% female; *p* = 0.346); baseline patient weight (81.5 kg [IQR 21.9 kg] vs. 84.2 kg [IQR 26.5 kg]; *p* = 0.494); prevalence of diabetes mellitus (27.1% vs. 40.3%; *p* = 0.138); and prevalence of hypertension (56.3% vs. 70.8%; *p* = 0.101).

### Incidence and source of infection

**Fig. 1:**
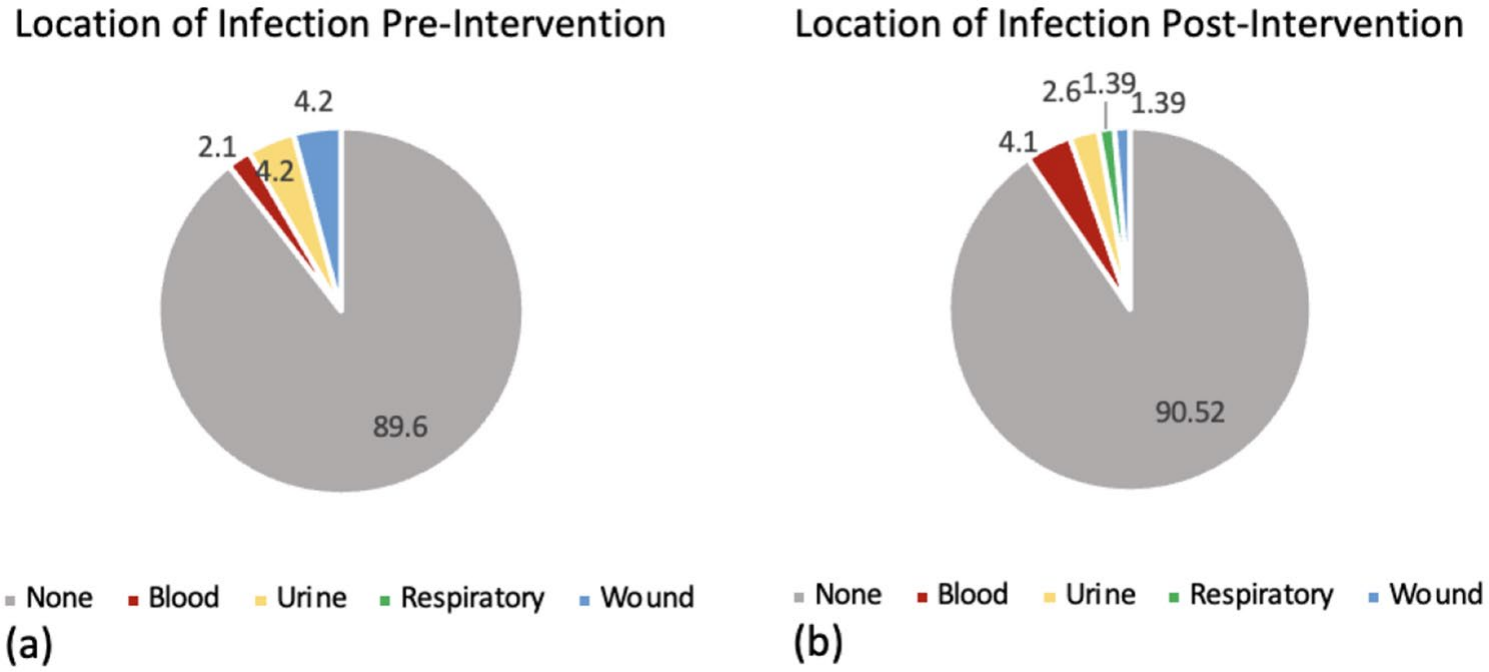
Source of infection in patients receiving (**a**) VPT (pre-intervention, *n* = 48) vs. (**b**) VC (post-intervention, *n* = 72), within 30 days post-OHT. Percentages displayed within figure. In the VPT group infections included: 1 blood, 2 urine, 2 respiratory. In the VC group, infections included: 3 blood, 2 urine, 1 respiratory, 1 wound

Investigating positive cultures amongst blood, urine, wound, and respiratory samples within 30 days of OHT, a total of 5 patients in the VPT group had positive cultures (10.4%) and 7 total patients in the VC group had positive cultures (9.7%). There was no significant difference in the rate of positive cultures among patients in the VPT group and those in the VC group (*p* = 0.781). Data on the pathogens and the location of infection are displayed in Table 1. No enterococcal organisms, specifically vancomycin-resistant enterococci (VRE), were cultured in either group (Table 1a). No significant difference was found in the location of infection among blood, urine, wound, and respiratory sources (*p* = 0.487) (Fig. 1a and b). Further, only one cultured organism was intrinsically resistant to the empiric antimicrobial treatment in the VC group, while none were intrinsically resistant in the VPT group (Table 1). In all but one case, the transplant infectious disease service was involved in the patient’s medical care and guided treatment decisions.

### Time to infection

**Fig. 2:**
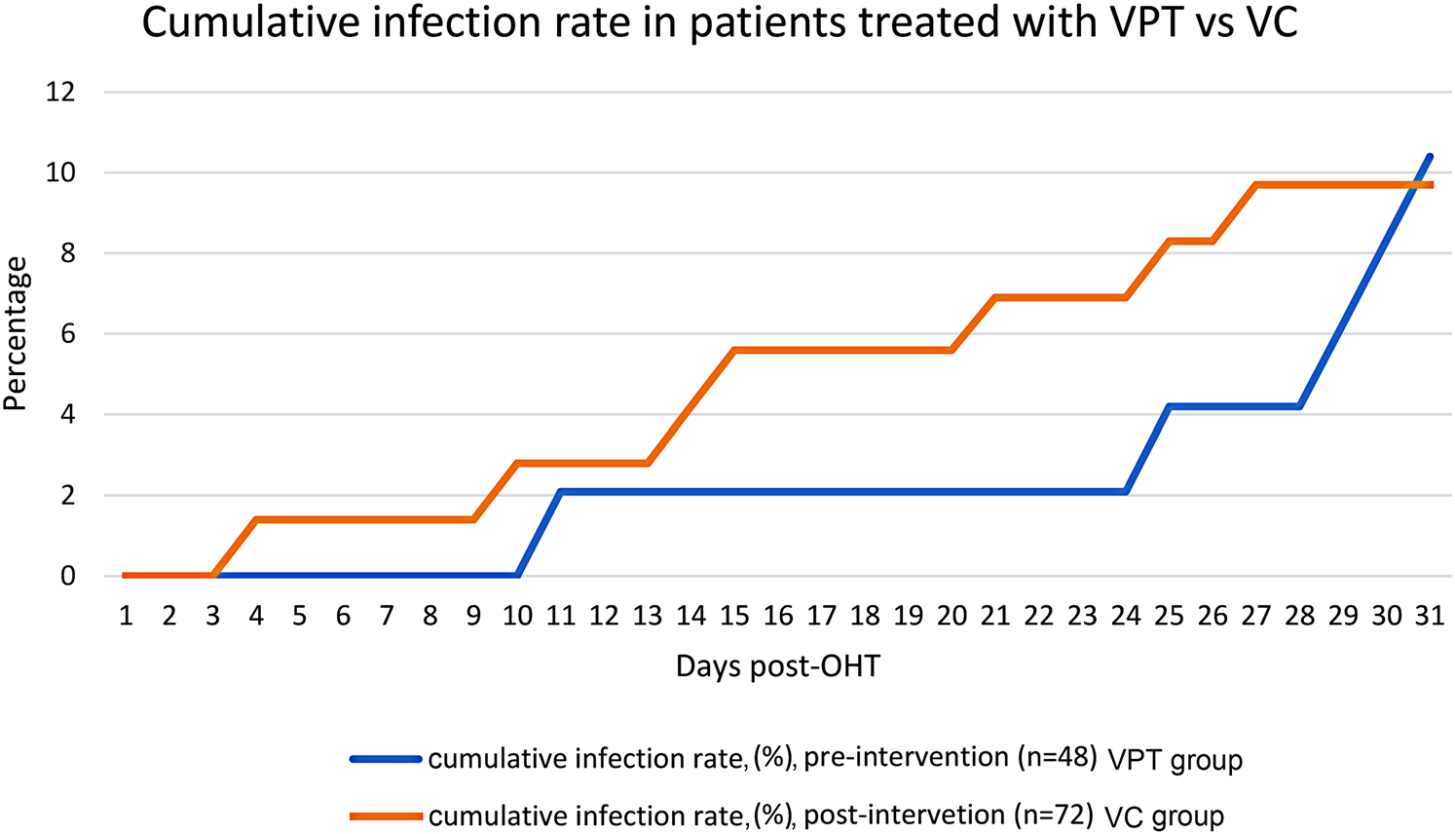
Time to infection post-orthotopic heart transplantation (OHT) and cumulative infection rate in patients treated with vancomycin and piperacillin-tazobactam (VPT) (*n* = 48) (blue line) vs. vancomycin and cefepime (VC) (*n* = 72) (orange line) within 30 days. Median time to infection in the VPT group was 28 days (IQR 5 days), and median time to infection in the VC group was 14 days (IQR 11 days)

Among patients with a culture-positive infection, time to event analysis showed a trend toward longer time to infection in the VPT group compared to the VC group. The median time to infection post-OHT was 28 days (IQR 5 days) in the VPT group versus 14 days (IQR 11) in the VC group; however, this difference failed to attain statistical significance (*p* = 0.08). While at no point was there a statistically significant difference in the cumulative number of infections between groups, the largest difference in the cumulative number of infections was at POD23 when the VPT group had 1 infection and the VC group had 5 infections (*p* = 0.40) (Fig. 2).

### Postoperative antimicrobial use

Within 7 days of OHT, 29.2% of patients were continued on an antimicrobial beyond the single intraoperative dose. In the VPT group, 22.9% of patients were continued, and in the VC group, 33.3% continued (*p* = 0.305). Coverage varied from broader coverage, including the continuation of VPT or VC, to narrower coverage with a single agent such as vancomycin, piperacillin-tazobactam, or penicillin G. Continuation was not standardized and reflected factors aside from culture positivity. This included clinical suspicion for infection and the presence of an open chest prior in cases of delayed sternal closure.

## Discussion

Piperacillin-tazobactam in combination with vancomycin is a frequently used empirical antibiotic regimen in hospitalized patients due to the broad spectrum of activity and is commonly used as antimicrobial prophylaxis in patients undergoing OHT. The risk of AKI from the combination of vancomycin and piperacillin/tazobactam has been well described in numerous observational studies and meta-analyses, resulting in an antibiotic change from VPT to VC or to another agent [5, 9]. In our institution, empiric intraoperative antimicrobial for OHT was changed from VPT to VC in an effort to decrease the rate of AKI post-OHT. However, our previously published data from this cohort failed to show evidence supporting the hypothesis that VPT increased rates of nephrotoxicity compared to VC in patients undergoing OHT [6].

Using the same cohort in this study, we sought to compare infectious outcomes in patients receiving VPT compared to those receiving VC. Specific antimicrobial choice depends on the surgical center, local susceptibilities, and patient population, among other variables. Prior studies and a large meta-analysis have suggested the use of cephalosporins to cover surgical site infection and respiratory infection, including ventilator-associated pneumonia, with vancomycin prophylaxis indicated for patient populations with a high rate of MRSA colonization [10, 11]. Prophylaxis beyond 48 h after any cardiac surgery has been shown to have no benefit [10], and studies are equivocal regarding continuation beyond the intraoperative period to 24–48 h. Patients in our study received standard intraoperative dosing of antimicrobial prophylaxis with a definitive change in protocol at our institution from VPT to VC, and this change allowed prospective comparisons of infectious outcomes.

Overall, our rates of microbiologically confirmed infection were similar to or lower than other studies, which report rates from 4.8% within 14 days of OHT to 35.9% during surgical admission at a median duration of 25 days [7, 12].

We report no significant difference in the rates of culture-positive infection between patients treated with VPT and those treated with VC. While this change resulted in the loss of targeted beta-lactam therapy for coverage of *Enterococcus* spp., vancomycin has coverage of *Enterococcus* outside of vancomycin-resistant enterococci (VRE), and no *Enterococcus* organisms were cultured from any patient. The change from VPT to VC also results in a loss of anaerobic coverage, with no culture-positive infections noted in either group; however, it is worth noting that these are uncommon post-transplant infections. A variety of organisms were cultured, with the most common being *Enterobacter* spp., with 1 in 4 infections attributable to this organism and including urinary, wound, and respiratory infections. Prior studies have shown variability in the most common pathogens, with two studies reporting the majority of infections post-OHT to be caused by *Enterobacterales* (of which *Enterobacter* is a member), another demonstrating more specifically *Escherichia coli* as the most common causative pathogen, while another reported methicillin- resistant *Staphylococcus* aureus [8, 13, 14].

Furthermore, we report no significant difference in the location of infection between patients receiving VPT and VC. Prior studies have demonstrated a higher incidence of infections from a respiratory source compared to other sources, accounting for 33% to 47% of infections [7, 8, 15]. As we used only microbiologically confirmed infection episodes, this may have contributed to a lower reported rate.

Limitations of our study include a relatively small patient population, few culture-positive infections, a follow-up period of only up to 30 days post-OHT, potentially not capturing all infections related to surgery, and the single-center design of the study. Future studies comparing outcomes between VPT and VC should be conducted in larger cohorts and in populations beyond our specific study population to validate these findings. Additional key outcomes of interest for comparison in future investigations include length of hospitalization, length of ICU admission, culture-negative febrile episodes, incidence of *Clostridiodes difficile* episodes, and incidence of toxic metabolic encephalopathy.

## Conclusions

Overall, our study demonstrates a relatively low rate of infection within 30 days of OHT in patients receiving empiric VPT or VC, with no significant difference in the location or the pathogens between groups. These findings suggest that both antibiotic combination regimens are viable options and are reassuring should a change to a less nephrotoxic antibiotic regimen be required to support implementation of the KDIGO Clinical Practice guideline for the management of AKI.

**Table 1:** Pathogen, source, antimicrobial resistance, and other details of infection in patients receiving VPT and VC

VPT, patients with post- operative infection (*n* = 5)	Positive culture location	Presumed source of infection	Vancomycin resistance	Piperacillin-tazobactam resistance	Duration of antibiotic therapy	Recurrence
Gram-positive organisms						
* Staphylococcus aureus*						
* Methicillin sensitive staphylococcus aureus (MSSA)*	Wound	Superficial surgical site wound infection	No		21 days	No
* Methicillin resistant staphylococcus aureus (MRSA)*	Blood	Parapneumonic effusion	No		14 days	No
Gram-negative organisms						
Klebsiella pneumoniae*	Urine	Catheter associated UTI		No	7 days	No
Enterobacter cloacae*	Urine	N/A		No	7 days	No
* Enterobacter spp*	Wound	Deep surgical site sternal wound infection with associated sternal osteomyelitis and pericarditis		No	6 weeks (post-sternal debridement, washout, flap)	No
Gram-negative organisms						
* Acinetobacter spp*	Blood	Deep surgical site wound infection (retrosternal collection)		No	14 days	No
* Burkholderia cepacia*	Blood	CLABSI (central line associated bloodstream infection)		Yes- intrinsically resistant to Cefepime	14 days	No
* Escherichia coli*	Urine	N/A		No	7 days	Yes
* Enterobacter aerogenes*	Wound	Groin wound (access site for CPB)		No	21 days	Yes
* Enterobacter cloacae*	Respiratory - BAL	Ventilator associated pneumonia		No	7 days	No
* Klebsiella pneumoniae*	Blood	Prior infection with IABP (remained in post-OHT)		No	7 days	No
* Escherichia coli* * Klebsiella pneumoniae* * Serratia marcescens*	Urine	N/A		E coli and klebsiella: no, Serratia: not performed	7 days	Yes, recurrent UTI with *E.coli*

*all urine cultures > 100k colony forming units speciated

## Supplementary Information

Below is the link to the electronic supplementary material.

Supplementary Material 1

## Data Availability

Data generated or analyzed for this study are available from the corresponding author on reasonable request.

## Abbreviations

AKI: Acute kidney injury
MRSA: Methicillin-resistant *Staphylococcus aureus*
MSSA: Methicillin-sensitive *Staphylococcus aureus*
OHT: Orthotopic heart transplantation
VC: Vancomycin and cefepime
VPT: Vancomycin and piperacillin-tazobactam
