# Supplementary material for: Infectious consequences of antimicrobial change from vancomycin-piperacillin/ tazobactam to vancomycin-cefepime in the prevention of AKI during orthotopic heart transplantation
Source: BMC Infect Dis. 2026 Mar 21;26:854. doi: 10.1186/s12879-026-12788-8 (PMC13130559; doi:10.1186/s12879-026-12788-8)
Supplement: Supplementary file 1 — Supplementary Material 1 [file 12879_2026_12788_MOESM1_ESM.docx]

Supplemental Figure 1

|  | Pre-Intervention (n=48) | Post-Intervention (n=72) | P – value |
| --- | --- | --- | --- |
| Vancomycin, median dose (g) | 1.25 | 1.25 | 0.259 |
| Vancomycin, median dose (mg/kg) | 14.99 | 14.89 | 0.529 |
| Piperacillin- tazobactam, median dose (g) | 18.0 | -- | -- |
| Cefepime, median dose (g) | -- | 4.0 | -- |

Total dosage of antimicrobials administered intra-operatively in patients undergoing OHT. In the pre-intervention group patients received vancomycin and piperacillin- tazobactam and in the post-intervention group, patients received vancomycin and cefepime. ***Piperacillin- tazobactam dosage was . Cefepime dosing was standardized as 2 grams IV (or renally dosed for patients with creatinine clearance less than 60 mL/min) then redosed every 4 hours for the duration patients remained in the operating room.
